# Integrated Characterization of *Phoenix dactylifera* L. Fruits and Their Fermented Products: Volatilome Evolution and Quality Parameters

**DOI:** 10.3390/molecules30143029

**Published:** 2025-07-19

**Authors:** Eloisa Bagnulo, Gabriele Trevisan, Giulia Strocchi, Andrea Caratti, Giulia Tapparo, Giorgio Felizzato, Chiara Cordero, Erica Liberto

**Affiliations:** Department of Pharmaceutical Science and Technology, University of Turin, Via Pietro Giuria, 9, 10125 Turin, Italy

**Keywords:** Dates (*Phoenix dactylifera* L.), functional fermented beverages, volatilome evolution, quality parameters, circular economy

## Abstract

Dates (*Phoenix dactylifera* L.) are nutrient-rich fruits with health-promoting properties and broad applications in the food and beverage industries. This study analyzes the chemical properties and volatile profile of fermented date products—juice, alcoholic derivative, and vinegar—to develop a high-quality vinegar with distinct sensory traits. Using HS-SPME-GC-MS, about 50 volatile compounds were identified across six major chemical classes. Juice processing significantly increased volatile release, especially fusel alcohols and furanic aldehydes, due to thermal and mechanical disruption. Fermentation further modified the volatilome, with increased esters and acids in alcoholic and vinegar products. Vinegar was characterized by high levels of acetic acid, fatty acids, phenols, and acetoin (855 mg/L), indicating active microbial metabolism. Ethanol and acidity levels met international standards. Total phenolic content rose from juice (138 mg/L) to vinegar (181 mg/L), reflecting microbial enzymatic activity and acid-driven extraction. These results highlight the metabolic complexity, sensory richness, and functional potential of date-derived fermented products while promoting sustainable use of underutilized fruit resources.

## 1. Introduction

The fruits of the date palm (*Phoenix dactylifera* L.) are a traditional and widespread food source in Southwest Asia and North Africa. They have exceptional nutritional, health, and economic value, which can be used not only for direct consumption in the form of dried or fresh fruit, but also for by-products such as energy bars, syrups, pastes, jams, desserts, sauces, and pickled dates [1,2]. Other products of fermented dates are alcoholic beverages such as wine, beer and liquor, and non-alcoholic beverages such as vinegar and juices [3]. Historically, dates have served both as a source of natural sweetness and nutritional value and as a functional substrate in the production of vinegar [3]. Scientific research and global market interests are increasingly driving towards healthy food and lifestyle (always referring to the Western world). According to various market analyses, global fermented foods and beverages are posed to acquire an important position in the food industry. During the forecast period between 2019 and 2026, this market is expected to grow at a compound annual growth rate (CAGR) of 8.1% [4,5,6,7]. As a result, different foods and beverages have been introduced to the market that have many expansion opportunities as products with improved properties. In the fruit sector, as well as others, the waste from the fruit business can be used in the production of brews and vinegars [8]. Date palm fruits are a flexible raw material, rich in nutritional elements and bioactive molecules such as carbohydrates, dietary fiber, polyphenols, carotenoids, and minerals. The high content of carbohydrates such as sucrose, fructose, and invert sugars (monosaccharides or disaccharides), which can make up to 75% of the dry weight of the fruit, lends itself to fermentation processes and can therefore be used to produce a variety of fermented beverages. Fermentation and enzymatic processing techniques form the basis of several bioprocessing strategies for the valorisation of date palm fruits and waste. The latter could be used as a raw material in the food industry to create high-sugar syrups that are used to produce both alcoholic and non-alcoholic fermented beverages, through the use of mainly lactic acid bacteria (LAB), yeasts, and acetic acid bacteria (AAB) [9,10,11,12,13,14]. A substantial proportion of low-ripening dates is discarded each year due to factors such as physical damage, unappealing morphology, or insufficient ripeness and size. There is a financial loss when low-grade secondary dates are not used. Consequently, efforts were made to develop intermediate date-based food products as functional co-products for industrial use, embracing the circular strategy and sustainable economy [12,13].

The sensory qualities of a product such as aroma, taste, and color are linked to its chemical composition. Volatile compounds influence flavor perception and complexity, while phenolic compounds affect taste (bitterness, astringency), color, body, mouthfeel, and aging [14,15]. The chemical composition defines the drink’s identity and is influenced by the fruit, ripeness stage and fermentation process. The purpose of the research was to characterize the HS-SPME-GC-MS compositional profile with regards to different palm date derivatives (juice, alcoholic derivative, and vinegar), enabling us to understand the chemistry behind the evolution of volatiles through the fermentative process. In parallel with the chromatographic profiling, the quantification of several quality markers was also carried out such as total and volatile acidity, ethanol, acetoin, and total polyphenolic content.

## 2. Results and Discussion

### 2.1. Volatilome of Different Palm Date Products

To preserve the compositional complexity and analytical integrity of volatile compounds associated with palm date matrices (fruit, juice, alcoholic, and vinegar phases) throughout the fermentation process, sampling protocols and instrumental conditions for headspace volatile collection were optimized to ensure adequate sensitivity and broad-spectrum recovery of key aroma-active constituents relevant to sensory characterization. Within detectable volatile analytes, about 50 of them were putatively identified in date products; they are listed together with retention times, IT, target and qualitfier ions, normalized responses, and odor quality in Table 1.

**Figure 1:**
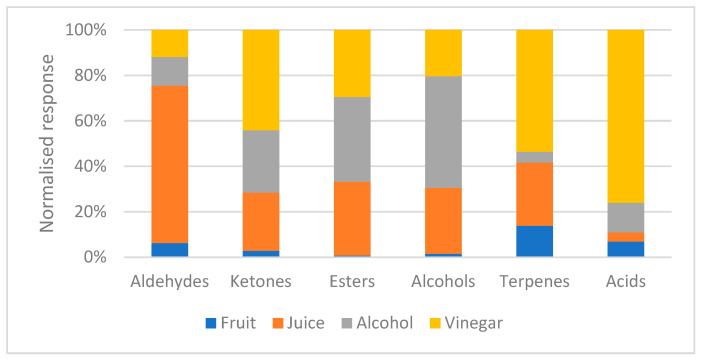
Comparison of the different chemical classes that constitutes the volatile fraction of the different palm dates’ products.

Palm dates are generally characterized by a relatively low abundance of volatile organic compounds. In the present study, six major chemical classes of volatiles were identified across the samples: aldehydes, ketones, esters, alcohols, terpenes, and organic acids (Figure 1). The processing step involving the transformation of whole fruits into juice led to an approximate fivefold increase in total volatile emission. This enhancement is attributed to mechanical disruption (shredding) and thermal treatment, which facilitate cellular rupture and the subsequent release of volatiles previously bound to or entrapped within the fibrous matrix.

**Figure 2:**
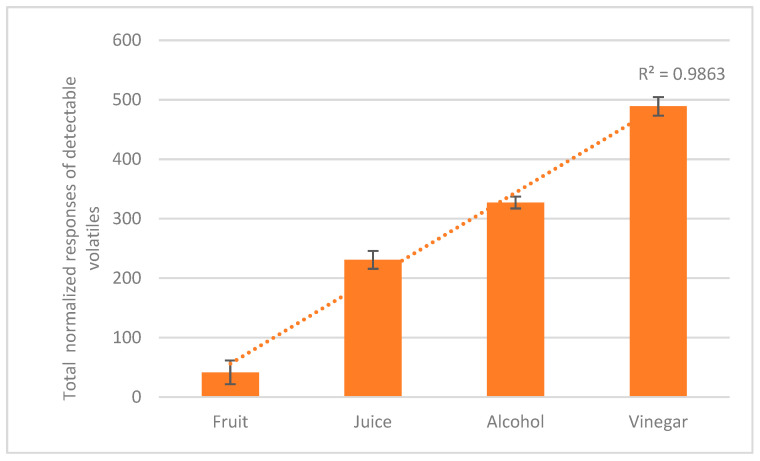
The volatilome’s richness of the different palm date product derivatives.

The volatilome of the further processing steps is instead the result of the fermentation effect on date juice. Nevertheless, the modification of the volatilome can be observed during processing, showing the linear evolution of the volatile components and their ratios as an expression of the metabolic effect of the co-operative microorganisms Figure 2.

**Figure 3:**
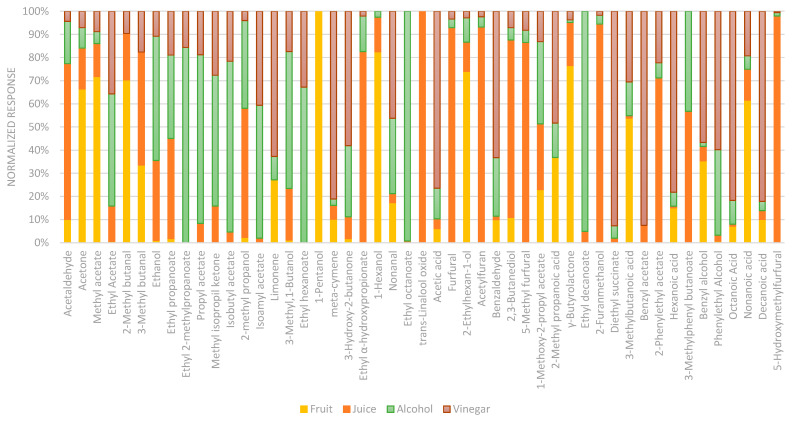
Comparison of volatile normalized area response of different matrices analyzed by HS-SPME-GC-MS.

In the date palm fruit, the development of aromatic compounds is linked to the botanical origin and ripening stage. At the Tamer stage (final maturation), alcohols and esters are predominant, with high levels of alcohol being particularly notable [16]. In fact, during fruit ripening, alcohols derived from amino acid metabolism were found to be the most important chemical class. Fusel alcohols (i.e., aliphatic alcohols with more than two carbon atoms) are produced via the Ehrlich pathway, where α-ketoacids produced by the initial transamination reaction are subsequently decarboxylated into aldehydes, which are then reduced into alcohols [6]. Compared to the other matrices, the abundance of 1-pentanol, 1-hexanol, and 2-ethyl-1-hexanol confirms this data in Figure 3 [14]. The palm fruit has a high content of lactones, correlated with the presence of butyrolactone, an element which then dissolves with fermentation, and of the terpene class in which limonene stands out. In the analyzed fruits, the presence of terpenes provides us with some more information on the derivation of the product since many other derivatives do not have it [16]. Another important group of compounds are the esters, such as methyl acetate and 1-methoxy-2-propyl acetate. However, their presence is lower than one might expect from the literature. In fact, date palms show a large development of ethyl acetate, ethyl octanoate, or ethyl decanoate. These compounds were not detected in the chromatographic profiles of the analyzed samples, which may be attributed to the specific characteristics of the fruit matrix and the nature of the comparison. In the literature, comparisons are typically made among different date palm cultivars, whereas in this case, the contrast involves distinct matrix types. Overall, the concentration of these compounds appears to be relatively low in fruit matrices compared to other substrates.

**Figure 4:**
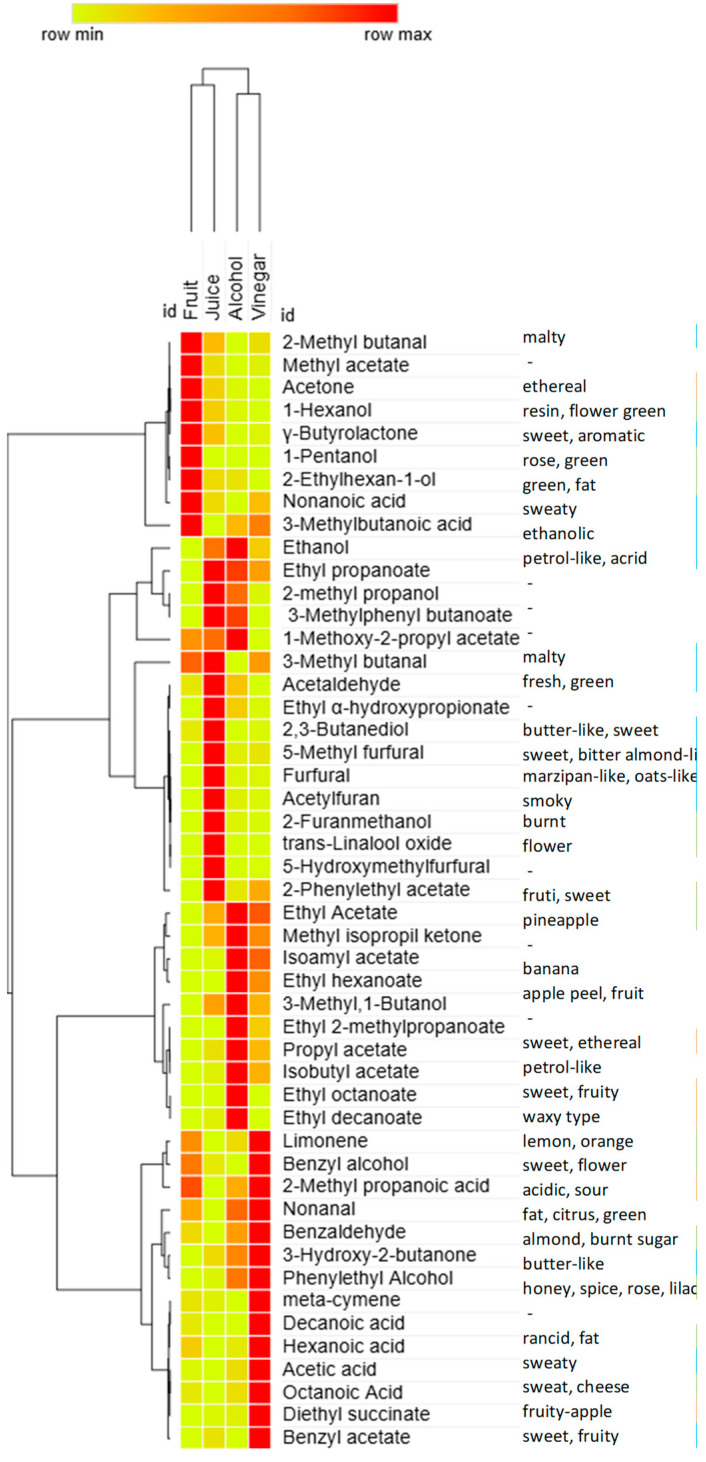
The evolution of the date’s aroma throughout fermentation process to vinegar (see Table 1).

Among the evaluated matrices, juice demonstrated a greater capacity to retain volatile compounds, likely due to the limited progression of fermentation. Compared to the native fruit, the juice exhibited increased concentrations of several volatiles, including acetaldehyde, ethyl acetate, 2,3-butanediol, selected fusel alcohols, and furanic aldehydes. Notably, 2,3-butanediol—one of the dominant volatiles in the juice—is a known bacterial metabolite typically formed during the early stages of fermentation. In addition, esters such as ethyl acetate and isoamyl acetate were detected, along with elevated levels of furan-derived compounds, particularly 5-hydroxymethylfurfural (HMF) and 2-furanmethanol. These latter compounds are likely products of thermal degradation of ascorbic acid (vitamin C) [17]. However, in this case, they are most probably formed during the thermal treatment before filtration. Given the time during which the fruits are boiled during thermal processing, furfural could be generated via the caramelization of fructose rather than Maillard reaction of fructose/glucose with aminoacids as reported from [18]. Trans-linalool oxide is present only in juice (Figure 3 and Figure 4) as the potential result of the biotransformation of linalool to pyranoid from fungi or derived from enzyme-catalyzed reactions [19]. In these cases, the enzyme lipase catalyzes epoxidation of double bonds analogous to the chemical reaction with a peroxycarboxylic acid [20].

Subsequently, in fermented products such as alcohol and vinegar, many compounds are lost due to the fermentation process, which leads to a decrease in the volatiles characteristic of fruit by converting these into other chemical classes such as esters and volatile acids. In general, higher values of ethanol and acetic acid are observed due to the fermentation process in which AAB converts glucose into ethanol and then into acetic acid [21]. The high percentage of esters in this matrix derived from the condensation between acetic acid and alcohol compounds, previously increased in alcoholic fermentation. During the aging of fermented beverages, due to alcohol fermentation and the reaction of acids with alcohols, the last ones go against a gradual decrease, converting into esters like isoamyl acetate, 2-phenylethyl acetate, and diethyl succinate [22]. As acetification progresses, acetic acid bacteria metabolize other alcohols, converting them into their corresponding acids. This process leads to a decrease in 3-methyl-1-butanol, 2-methyl-1-butanol, and 1-hexanol, while octanoic, decanoic, and hexanoic acids increase, as shown in Figure 3 and Figure 4. The heatmap in Figure 4 visually highlights the volatilome evolution at the different stages of production of palm date products. Based on Euclidean distances, the data are grouped into two hierarchical clusters: one containing fruit and juice and another with alcoholic and vinegar. Different colors indicate the significance of chemical compounds, with yellow representing a lower and red indicating higher abundance of volatile compounds. The relative amount of volatile compounds varies according to the fermentation process, showing a gradual increase from fruit (yellow) to juice and finally to vinegar. Acidification decreases ethyl esters due to hydrolysis, influenced by acidification duration and bacteria used [23]. Acetoin (3-hydroxy-2-butanone), considered one of the most aroma-active compounds, exists in some foods, especially in vinegars as a metabolite produced by *Lactobacillus* and *Acetobacter* during acetic acid fermentation [24]. Its presence can impart buttery, fruity, or caramel notes, adding depth to the sensory profile of the vinegar. However, an excess of acetoin may indicate incomplete fermentation or a suboptimal production process, negatively impacting the vinegar’s quality. This compound was evaluated first because its presence is an indication of authenticity; second, because its concentrations give us indications about the characteristic and type of products. Date vinegar presents a significantly high concentration of acetoin (855 ± 150 mg/L) that is an important aromatic compound produced during acid fermentation by bacteria such as *Lactobacillus* and *Acetobacter*. However, unlike wine vinegar in which the determination of acetoin is used for authenticity and quality and its content should be between 100 mg/L and over 400 mg/L, no reference is reported in the literature about the acetoin amount in date vinegar [25,26]. The production of acetoin is influenced by the combinations of bacterial strains used, which can vary among different date fermented beverages. Additionally, the oxidation of 2,3-butanediol by *Acetobacter* contributes to the high acetoin content in date vinegar [27]. In support of this relationship, a negative correlation of −0.453 was observed between 2,3-butanediol and acetoin in the analyzed samples. Although moderate in strength, this inverse association suggests a potential metabolic interconversion between the two compounds.

### 2.2. Ethanol Contents and Acidity

The analysis of ethanol and total volatile acidity is crucial for assessing the quality and legal compliance of fermented beverages. Dates and their derivatives, such as date vinegar, exhibit variable acidity levels depending on production methods and the maturity of the fruit used. Compared with other fermented beverages, it emerges that production methods, regional specifications, and the management of additives play a significant role in determining the final acidity of the product.

The samples underwent submerged fermentation, an aerobic fermentation process that uses a powerful aeration system to supply the system’s oxygen requirement while suspending acetic acid bacteria in the acetifying culture [21]. Strong acetic acid bacteria cultures that can convert ethanol into acetic acid are necessary for this aerobic fermentation; successful fermentation also depends on the right temperature and oxygen availability.

The amount of ethanol found in the alcoholic date derivative was 5.82%. The values recorded in our study are consistent with those reported for date palm matrices, where ethanol content typically ranges from 4.26% to 6.78% *v*/*v* after 96 h of fermentation [28]. Due to the fermentation process, vinegar samples have very low alcohol content; in fact, ethanol oxidizes to produce molecules of acetic acid. Residual ethanol and acidity are two main parameters evaluated to classify vinegar that has different definitions and characteristics depending on the specific country’s regulation. FAO/WHO defines vinegar as “…a food derived from a source rich in sugar and starch that underwent to a double fermentation alcoholic and acetous with an ethanol content less than 0.5% in wine vinegar and less than 1% in other vinegars….” [29]. In the USA, there are no official standards of identity for labeling various types of vinegar, including cider, wine, malt, sugar, spirit, and blends; the only requirement from the FDA is that vinegar products have a minimum acidity of 4% to ensure retail strength. In China, vinegar has only recently been classified as natural fermented product or as artificial, i.e., acetic acid mixed with other ingredients; previously the term “vinegar” included both with a different set of acetic acid from 3.5% to > 6%. European countries follow regional standards for vinegar that is produced or marketed within their territories [30]. In contrast to other countries, the European Union has set specific limits for both acidity and ethanol content. The term ‘Vinegar of X’ broadly refers to products containing at least 5% (*w*/*v*) acidity and no more than 0.5% (*v*/*v*) ethanol. Wine vinegar, which must be derived exclusively from the acetous fermentation of wine, is required to have a minimum acidity of 6% (*w*/*v*) and may contain up to 1.5% (*v*/*v*) ethanol, in accordance with law [31].

In Europe, vinegar is mainly used as a sharp, sour flavoring or preservative. In contrast, in Asia and Africa, it is also consumed as a milder-tasting drink, with sweetened, low-acidity fruit vinegars especially popular in China and Southeast Asia. In Europe’s wine-producing regions, clear distinctions exist between wine and vinegar, with well-established terminology. However, in Africa, some fermented beverages naturally acidify into alcoholic-acetous products, though they are difficult to classify. In Japan, black rice vinegar is often diluted with fruit juice and consumed as a health tonic. However, under EU and FAO/WHO definitions, many fruit vinegars do not qualify as true vinegar due to low acetic acid and residual ethanol.

The ethanol level in the vinegar date samples, measured at 0.39% *v*/*v*, is also within the acceptable limit (<0.5% *v*/*v*) defined for commercial vinegar in EU [25]. The results obtained align with the literature, confirming the low alcohol content in vinegar samples as a consequence of ethanol oxidation into acetic acid during the fermentation process [21].

Scientific research has limited references for date matrices, although some values are provided for date vinegar [28]. In the Matloob study, the juice was mixed with old vinegar and used as the starting material for vinegar production, developing an acidity between 6.50% and 7.28% *w*/*v*. For Iraqi date vinegar, after the addition of unpasteurized old vinegar to start fermentation, a value of about 4.02% *w*/*v* was obtained. Our vinegar samples contain an average of 6±0.1% *w*/*v* of acidity.

### 2.3. Polyphenols

Phenolic compounds vary significantly between date species, cultivars, climatic conditions, and ripening stage [12]. Polyphenols are essential bioactive constituents of palm date juice, wine, and vinegar owing to their nutritional and sensory contributions. In palm date juice, polyphenols enhance the functional beverage properties and provide antioxidant and anti-inflammatory benefits [32]. In wine, they impact its color, flavor, astringency, while also protecting cardiovascular and metabolic health [33]. In the process of producing vinegar, especially from fruits and wines, polyphenols are either retained or transformed, thereby augmenting the final product’s antioxidant capacity, antimicrobial activity, and health-promoting benefits. The overall shelf life and functional value of these products have improved due to their presence. Studies have reported total phenolic contents ranging from 281.17 mg GAE/L to 641.17 mg GAE/L with significant differences (*p* < 0.05) noted between homemade and commercial date vinegar [34,35]. Our samples showed an increase in the total phenolic content from the palm date juice to vinegar, respectively, 138 ± 1.95 mg/L in juice, 152 ± 1.56 mg/l in the alcoholic derivative, and 181 ± 0.78 mg/L in palm date vinegar. Although the literature is not always aligned, generally an increase in phenolic content is observed during fermentation [9,34,36]. The increase in total phenolic content is due to a breakdown of cell walls from microbial enzymes releasing bound polyphenols in which solubility and stability is enhanced from the acidification. This phenomenon is still occurring during the submerged fermentation kept under agitation and heating in which polyphenols continue to leach out from solids over time [36,37]. These polyphenols contribute to the antioxidant properties of date vinegar, which have been associated with potential health benefits such as reducing cholesterol levels and inflammation [21,32,33].

## 3. Materials and Methods

### 3.1. Samples

Palm date samples and three samples of spontaneously fermented date beverages (juice, alcohol, and vinegar; two for each matrix) were provided by Ponti s.p.a. [Ghemme (NO), Italy]. Date juice was prepared by boiling a mixture of dates and water in a 1:3 ratio for 5 min, followed by filtration. The main characteristics of this product are a pH ranging from 5.54 to 6.43 and a Brix degree varying from 17.4 to 20.6. The other products were fermented with a mixture of date alcohol and date vinegar as a starter, trying to maintain a balance of 6% alcohol and 4% acidity. After 7 days of static adjustment, submerged fermentation was maintained for 17 days and then stopped. Once samples were received, they were stored in glass jars, avoiding headspace, and kept in a fridge at 5 °C to slow down the fermentation process.

### 3.2. Standard References and Solvents

The homologous series of n-alkanes (from n-C_9_ to n-C_25_) for linear retention index (*I*^T^_S_) calculation; n-C_13_ as internal standard (ISTD); Folin–Ciocâlteau reagent, gallic acid, sodium carbonate to establish polyphenolic content, acetoin, sodium hydrate (0.25 N), dibutyl phthalate (99%), ethanol (96%), and methanol (100%) were all obtained from Merck (Merck, Milan, Italy).

### 3.3. Volatile Profiling

**Figure 5:**
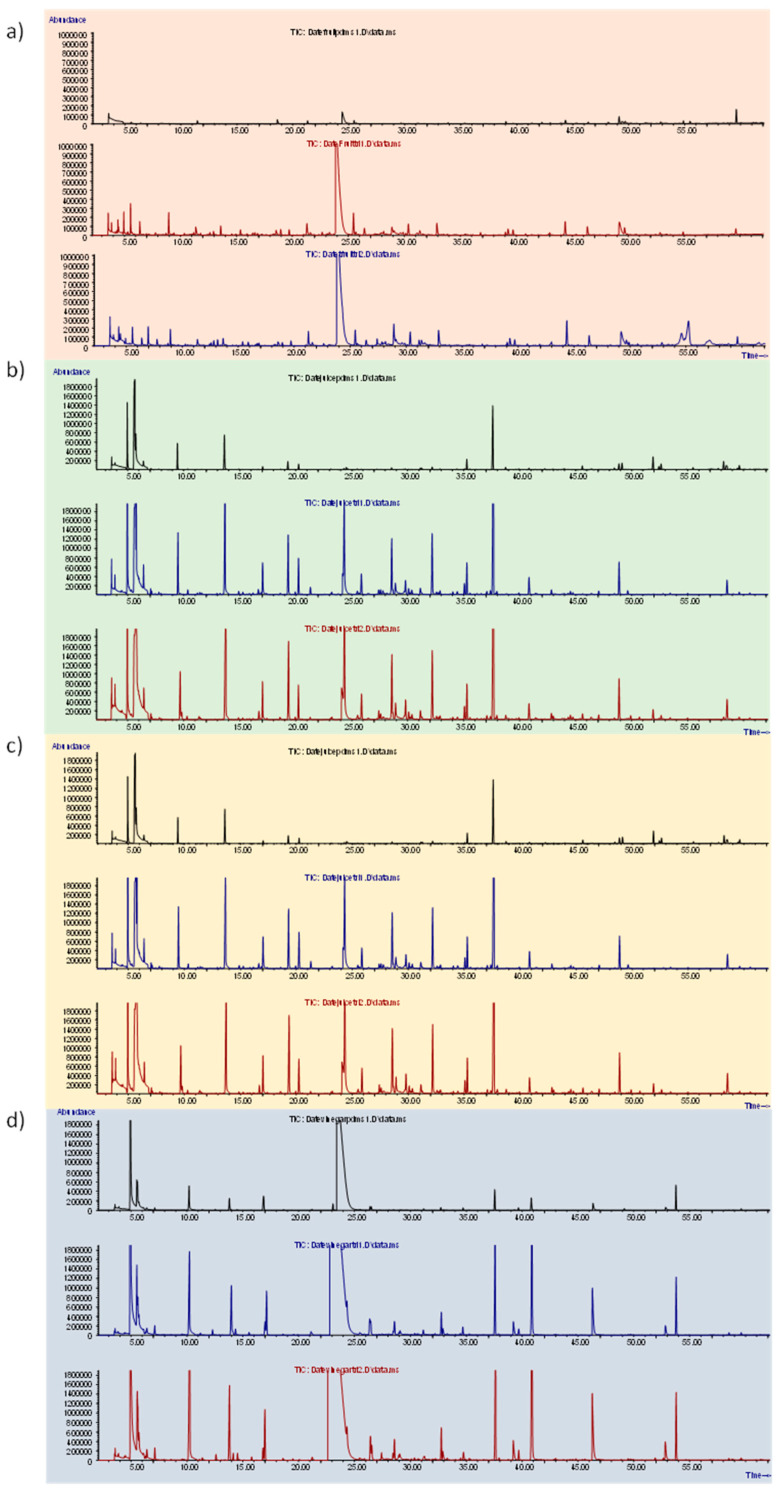
Comparison of chromatographic profiles of HS-SPME sampling with different polymeric coatings on (**a**) fruit, (**b**) juice, (**c**) alcohol, (**d**) vinegar.

Volatiles were sampled using an automatic HS-SPME system installed on an MPS-2 multipurpose sampler controlled by a Gerstel Maestro software version 1.4.6.5 (Gerstel, Mülheim a/d Ruhr, Germany) online combined with the GC-MS instrument (see details below). In total, 3 g of date fruit, 6 g of juice, 5 mL of wine, and 5 mL of vinegar were separately weighed in 20 mL vials; the amount of date products was defined in order to have the same phase ratio (β) within the vial. Then, the vials were hermetically closed with a polytetrafluoroethylene septum. Before use, the fiber was conditioned according to the manufacturer’s instructions. The choice of the polymeric coating was made with reference to the literature by performing experimental comparison at 50 °C for 30 min between PDMS and DVB/CAR/PDMS (1 cm and 2 cm length) fibers from Supelco (Bellefonte, PA, USA). The comparison between the different polymeric coatings was made on all the different date matrices (fruit, juice, alcohol, and vinegar). Results showed similar performances in volatiles extraction for the DVB/CAR/PDMS coating slightly higher with the 2 cm fibers (Figure 5). The coating divinylbenzene/carboxen/polydimethylsiloxane (DVB/CAR/PDMS) was therefore used for the extraction of the volatile fraction of the different samples. Sampling was performed at 50 °C for 30 min with the addition of ISTD (n-C_13_) to normalize responses among analytical runs. The n-C_13_ (Rt: 15.42 min) was chosen as an internal standard because it was not pre-sent in the profiles during the fibers’ coatings screened as shown in Figure 5. The standard-in-fiber procedure was adopted to pre-load the ISTD onto the fiber before sampling; 5.0 µL of ISTD solution at 1000 mg/L was placed into a 20 mL glass vial and submitted to HS-SPME at 50 °C for 5 min, stirring speed of 350 rpm [38].

### 3.4. Alcoholic Degree Determination

Two replicates of 500 µL palm date alcohol diluted 1:20 and the same for vinegar diluted 1:5 were evaluated to assess the ethanol content by the use of HS-SPME-GC-MS (extimated by an external calibration curve). The results were expressed as g/100 g. The stock standard solution of ethanol was prepared at 50,000 mg/L. Working solutions were obtained by diluting this stock to 1500; 1000; 750; 500; 250; and 100 mg/L. The content of ethanol was expressed as % *w*/*v*.

### 3.5. GC-MS Instrument Setup

The GC–MS system consisted of an Agilent 7890A GC coupled with an Agilent 5975B MS detector (Agilent, Little Falls, DE, USA) operating in EI mode at 70 eV. The injector temperature was set at 250 °C in split mode, split ratio: 20:1. A SolGel-WAX^®^ column (100% polyethylene glycol, 30 m × 0.25 mm dc, 0.25 μm df) from Trajan (Trajan, Melbourne, Australia) was used. The carrier gas was helium at a constant flow rate of 1 mL/min. The oven temperature program was 40 °C (3 min) to 200 °C at 3 °C/min, then to 200 °C at 5 °C/min and finally to 250 °C (5 min) at 10 °C/min. The GC transfer line was set at 270 °C, and the ion source temperature at 230 °C. The scan range was set to 35–350 m/z with a scan speed of 666 amu/s. Data were acquired with Agilent MSD Chemstation E.02.01.1177 software (Agilent Technologies). Volatile components were identified by comparing their calculated linear retention index (*I*^T^s) and mass spectra with those collected in in-house and/or commercial databases (Wiley 7N and NIST 14 Mass Spectral Data) or reported in the literature, and with an authentic standard available.

### 3.6. Total and Volatile Acidity Content Determination

Acidity is an important organoleptic parameter for determining certain characteristics and is divided into total and volatile acidity. Total acidity is the sum of fixed acids and volatile acids. Volatile acidity is an indicator of bacterial oxidation conditions. The procedure for total acidity involves the removal of CO_2_ through vacuum extraction; 25 mL of sample was then subjected to potentiometric titration at pH 7 with NaOH 0.25 N at 20 °C. Meanwhile, the procedure for volatile acidity includes the removal of CO_2_ and subsequent steam distillation to extract volatile acids, followed by titration with NaOH 0.25 N and phenolphthalein as an indicator [39]. The alcoholic matrix of date palm alcohol was compared using the must method, with tartaric acid as the reference acid [40,41].

### 3.7. Acetoin Content Determination

Acetoin content was accurately determined by HS-SPME-GC-MS with the external calibration method. Five hundred microliters of vinegar was sampled and analyzed as reported in Section 3.3 and Section 3.5. The stock standard solution of acetoin was prepared at 5000 mg/L in water. Working solutions were obtained by diluting the stock solution to 1000, 750; 500, 250 mg/L.

### 3.8. Polyphenolic Content

The polyphenol content was determined through the Folin–Ciocâlteu method by means of an external calibration curve based on gallic acid. This is an approximate method in which results are usually expressed as the amount of gallic acid per weight of extract. Additionally, 1 mL of date matrix sample was added to 5 mL of milliQ water and 1 mL of Folin–Ciocâlteau reagent. The solution was stirred and finally 4 mL of 10% sodium carbonate was added and made up to volume with milliQ water (20 mL). After 90 min in dark conditions, the solution was analyzed by UV-Vis spectroscopy with a Thermo Genesys 6 spectrophotometer (Thermofisher, Rhône, France) in two replicates [42]. The quantification was at 750 nm and results are given as mg/L of gallic acid equivalents (GAE) from external calibration curve (R^2^ = 0.998). The stock standard solution of gallic acid was prepared at 1000 mg/L in methanol. Working solutions were obtained by diluting the stock solution up to 250; 100; 50; and 25 mg/L.

### 3.9. Statistical Analysis

The data were processed with Agilent MSD Chemstation E.02.01.1177 and Excel 2016. Heatmap was carried out with Morpheus (https://software.broadinstitute.org/morpheus/ Accessed on 3 April 2025).

## 4. Conclusions

This research has allowed for the characterization of the volatilome of palm dates and their fermented derivatives (juice, alcohol, vinegar) using the HS-SPME-GC-MS technique. The analysis of the chromatographic profile has provided a detailed understanding of the evolution of volatile compounds throughout the fermentation process. Key quality parameters, such as total and volatile acidity, ethanol, acetoin, and polyphenolic content, were carefully evaluated, contributing to the understanding of the chemical transformations occurring during the fermentation process. These findings not only enhance the knowledge of the aromatic and qualitative profile of palm date-derived products but also serve as important indicators for determining the quality and authenticity of these fermented foods The long history of date consumption and the widespread availability of this raw material in many regions of the world make it an attractive option for vinegar production, providing a natural and sustainable alternative to traditional vinegars, but also for new fermented drinks. The findings highlight the potential of using dates, including lower-grade fruit, to produce high-quality, functional vinegars—supporting sustainability and circular economy through the value-added utilization of underused resources.

## Figures and Tables

**Table 1:** Dates’ volatile compounds obtained by HS-SPME-GC-MS together with their retention time, experimental and literature I^T^s, target ion, qualifier ions, and chromatographic normalized response for each matrix.

Compounds	Rt (min)	I^T^ exp	I^T^ lit	Target Ion	Qualifier Ions	Normalized Response (vs. IS)				
						*Fruit*	*Juice*	*Alcohol*	*Vinegar*	*Odor Description ^1^* ^,*2*^
Acetaldehyde	1.86	715	724	44	45; 46	109.4	729	197.9	47.5	*fresh*, *green*
Acetone	2.33	820	821	43	58; 42	46	12.2	6.1	4.9	*ethereal*
Methyl Acetate	2.39	836	832	43	74; 59	83.2	16.5	6	10.2	*ethereal*
Ethyl Acetate	2.95	878	895	43	61; 70	76.9	2188.7	6921	5186.3	*pineapple*
2-Methyl Butanal	3.24	898	942	57	58; 39	7.5	2.1	-	1	*malty*
3-Methyl Butanal	3.31	902	917	44	58; 39	7.1	10.3	-	3.7	*malty*
Ethanol	3.73	960	956	45	46; 47	225.2	7574.8	11,758.5	2373	*ethanolic*
Ethyl Propanoate	4.01	969	961	57	75; 74	0.7	16.6	13.8	7.3	*petrol-like*, *acrid*
Ethyl 2-Methylpropanoate	4.09	971	971	43	71; 45	-	-	131.9	24.4	*sweet*, *ethereal*
Propyl Acetate	4.33	978	977	43	61; 73	-	8	69.9	18.1	*petrol-like*
Methyl Isopropyl Ketone	4.38	980	989	43	86; 44	-	38.8	137.7	67.6	*camphor*
Isobutyl Acetate	5.14	1002	1002	43	56; 73	1.5	12.9	226.6	66.5	*fruit*, *apple*, *banana*
2-Methyl Propanol	7.67	1078	1078	43	73; 74	10	237.4	161.5	17.1	*Ethereal-type*
Isoamyl Acetate	8.28	1096	1107	43	70; 55	2	31.9	1027.6	689	*banana*
Limonene	10.1	1171	1180	68	67; 93	6.7	-	2.5	15.4	*Lemon*, *orange*
3-Methyl-1-Butanol	11.74	1200	1202	55	70; 39	39	747.1	1976.3	584	*fermented*, *fusel alcohol*
Ethyl Hexanoate	12.35	1214	1223	69	281; 53	-	-	24.6	12	*apple peel*, *fruit*
1-Pentanol	13.38	1242	1241	42	55; 70	23.1	-	-	-	*fermented*, *pungent*
*p*-Cymene	13.89	1255	1263	119	134; 91	2.6	1.5	0.7	20.6	*terpenic*, *citrus*
Acetoin	14.68	1275	1273	45	88; 44	39.7	206.8	675.5	1276.2	*butter-like*
1-Hexanol	17.68	1349	1351	56	55; 69	37.6	6.8	1.2	-	*resin*, *flower green*
Nonanal	19.24	1386	1385	57	56; 55	28	6.3	52.6	74.9	*fat*, *citrus*, *green*
Ethyl Octanoate	20.93	1428	1429	88	57; 101	-	3.2	441.5	-	*sweet*, *fruity*
*trans*-Linalool Oxide	21.17	1434	1432	59	94; 55	-	17.2	-	-	*Flower*
Acetic Acid	20.33	1413	1413	43	45; 60	2.5	1723.8	5209.7	31,295.5	*sour*
Furfural	22.28	1461	1462	96	95; 39	3	673.6	26.7	25.1	*marzipan-like*, *oats-like*
2-Ethyl,1-Hexanol	23.42	1488	1488	57	55; 70	69.7	11.9	9.9	2.7	*rose*, *green*
2-Acetylfuran	23.81	1498	1507	95	110; 43	-	534.3	25.6	13.6	*smoky*
Benzaldehyde	24.39	1512	1500	106	105; 77	23.9	3.6	60.3	151	*almond*, *burnt sugar*
2,3-Butanediol	25.35	1537	1539	45	57; 47	19.1	133.8	9.3	12.4	*butter-like*, *sweet*
5-Methylfurfural	26.52	1566	1567	110	109; 53	-	383.8	23.3	36.5	*sweet*, *bitter almond-like*
1-Methoxy-2-Propyl Acetate	26.85	1574	1570	43	88; 89	18.1	22.4	28	10.4	*-*
2-Methyl Propanoic Acid	27.01	1579	1578	43	73; 39	69.3	-	28	90.7	*acidic*, *sour*
γ-Butyrolactone	28.37	1613	1611	42	86; 56	290.1	70.6	4.4	14.2	*sweet*, *aromatic*
Ethyl Decanoate	29.05	1631	1639	88	101; 55	-	3.8	73.7	-	*sweet*, *fruity*
2-Furanmethanol	30.12	1659	1661	98	97; 53	0.8	642.7	25.9	12	*burnt*
Diethyl Succinate	30.69	1674	1677	101	129; 55	-	11.7	31.8	554.4	*fruity-apple*
3-Methyl Butanoic Acid	30.93	1680	1676	60	74; 87	1.6	3.3	44.3	93	*sweaty*
Benzyl Acetate	32.38	1719	1711	108	91; 90	-	0.7	-	8.6	*sweet*, *fruity*
2-Phenylethyl Acetate	35.58	1808	1811	104	91; 105	2.4	3778.7	346.9	1183	*Fruit*, *sweet*
Hexanoic Acid	37.28	1857	1865	60	73; 55	58	2.2	23.6	299.3	*sour*, *fatty*
3-Methylphenylbutanoate	37.62	1867	-	71	79; 108	-	10.3	7.9	-	*-*
Benzyl Alcohol	37.63	1867	1861	79	108; 107	16.1	2.8	0.8	25.9	*sweaty*
Phenylethyl Alcohol	38.75	1899	1894	91	92; 122	37.1	143.2	2072.9	3350.1	*honey*, *spice*, *rose*, *lilac*
Octanoic Acid	44.32	2068	2064	60	73; 101	6.8	7.6	98	777.1	*sweat*, *cheese*
Nonanoic Acid	47.57	2173	2164	60	73; 57	11.7	13.8	6	20	*green*, *fat*
Decanoic Acid	50.75	2279	2276	60	73; 129	9.6	3.6	3.8	78.2	*rancid*, *fat*
5-Hydroxymethylfurfural	56.38	2480	2485	97	126; 69	-	173.4	2.5	1.1	*fatty*, *buttery*

^1^ https://www.thegoodscentscompany.com/; ^2^ https://www.flavornet.org/flavornet.html. Accessed on 24 March 2025.

## Data Availability

Data is contained within the article.
